# Supplementary material for: Fast and accurate taxonomic domain assignment of short metagenomic reads using BBERT
Source: Nucleic Acids Res. 2026 Oct 22;54(19):gkag1002. doi: 10.1093/nar/gkag1002 (PMC13687566; doi:10.1093/nar/gkag1002)
Supplement: Supplementary file 1 [file gkag1002_supplemental_files.zip › BBERT Revision NAR 2026-07-25_only_SI.pdf]

Supplementary Figures

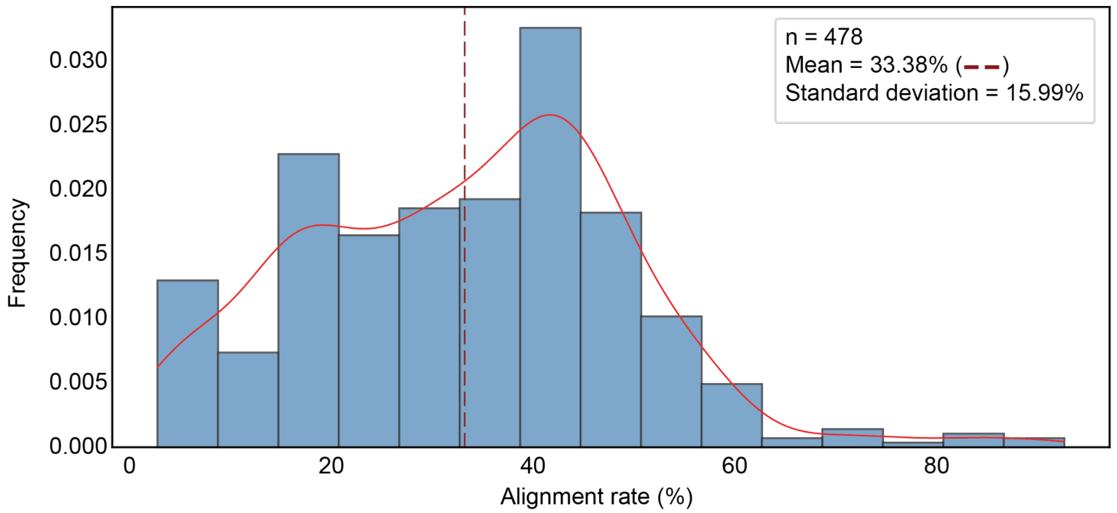

Fig. S1. Percent of reads mapped to assemblies of 478 soil metagenomes. The mean alignment rate is 33.38%, meaning that 66.62% of the reads do not align to any contig.

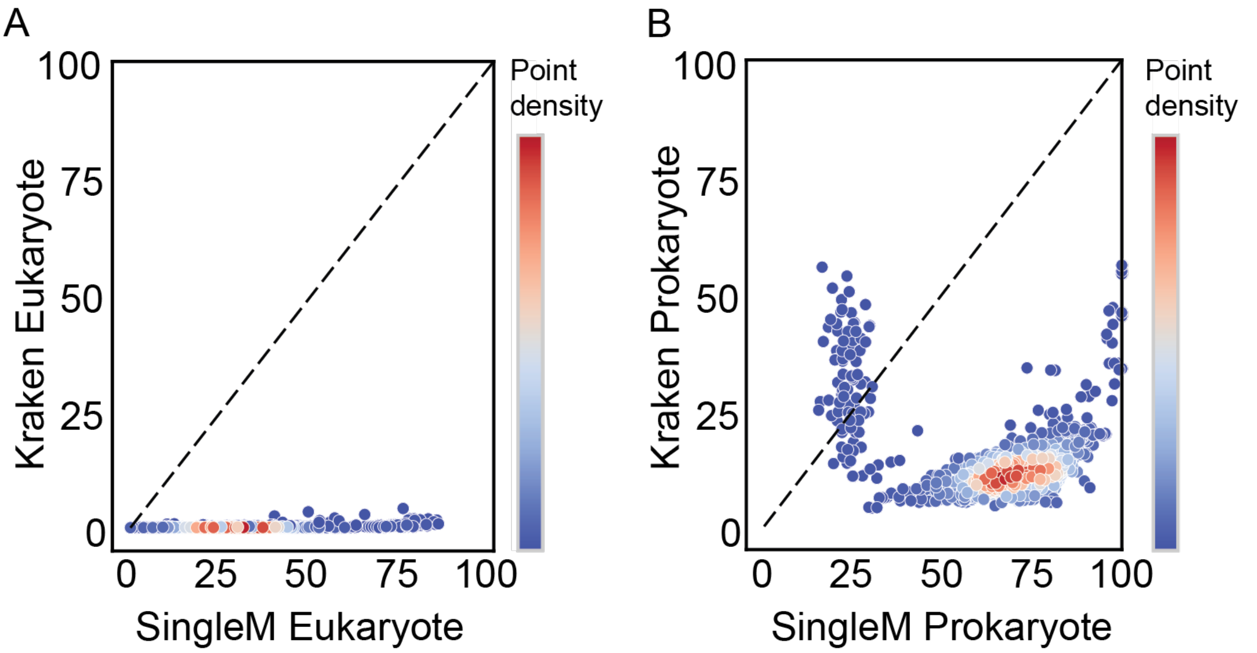

Fig. S2. Further evidence for conflicting eukaryote and prokaryote fractions between SingleM and Kraken2's default database. The imputed domain percent of each sample calculated using SingleM on the *x*-axis and Kraken2 using its default database plus fungi, protozoa, and yeast (PlusPFP, September 2024). **(A)** Imputed eukaryote fraction, where only positively identified eukaryotes are considered for Kraken2. **(B)** The imputed prokaryote percent of each sample.

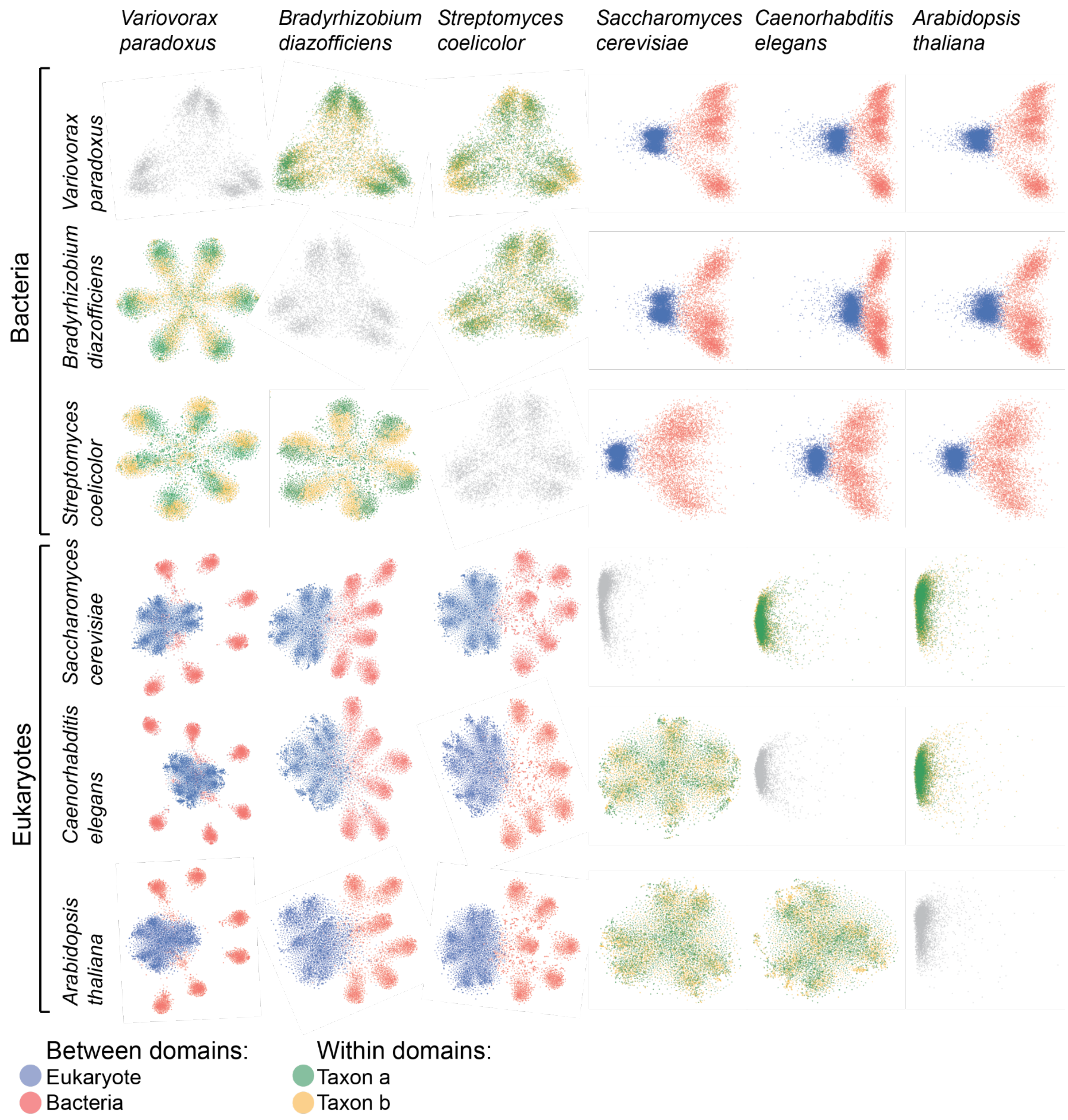

Fig. S3. Generalization of BBERT embedding structure across taxa within and between domains. Grid of pairwise taxon comparisons derived from per-read BBERT embeddings. Off-diagonal panels show joint embeddings of the two indicated taxa: the lower triangle uses t-SNE, and the upper triangle shows PCA (PC1 vs. PC2). Diagonal panels display single-taxon PCA to highlight within-taxon substructure. Across pairs, both methods robustly separate taxa, including across domains, while single-taxon views reveal finer organization consistent with content-based features (Fig. 2).

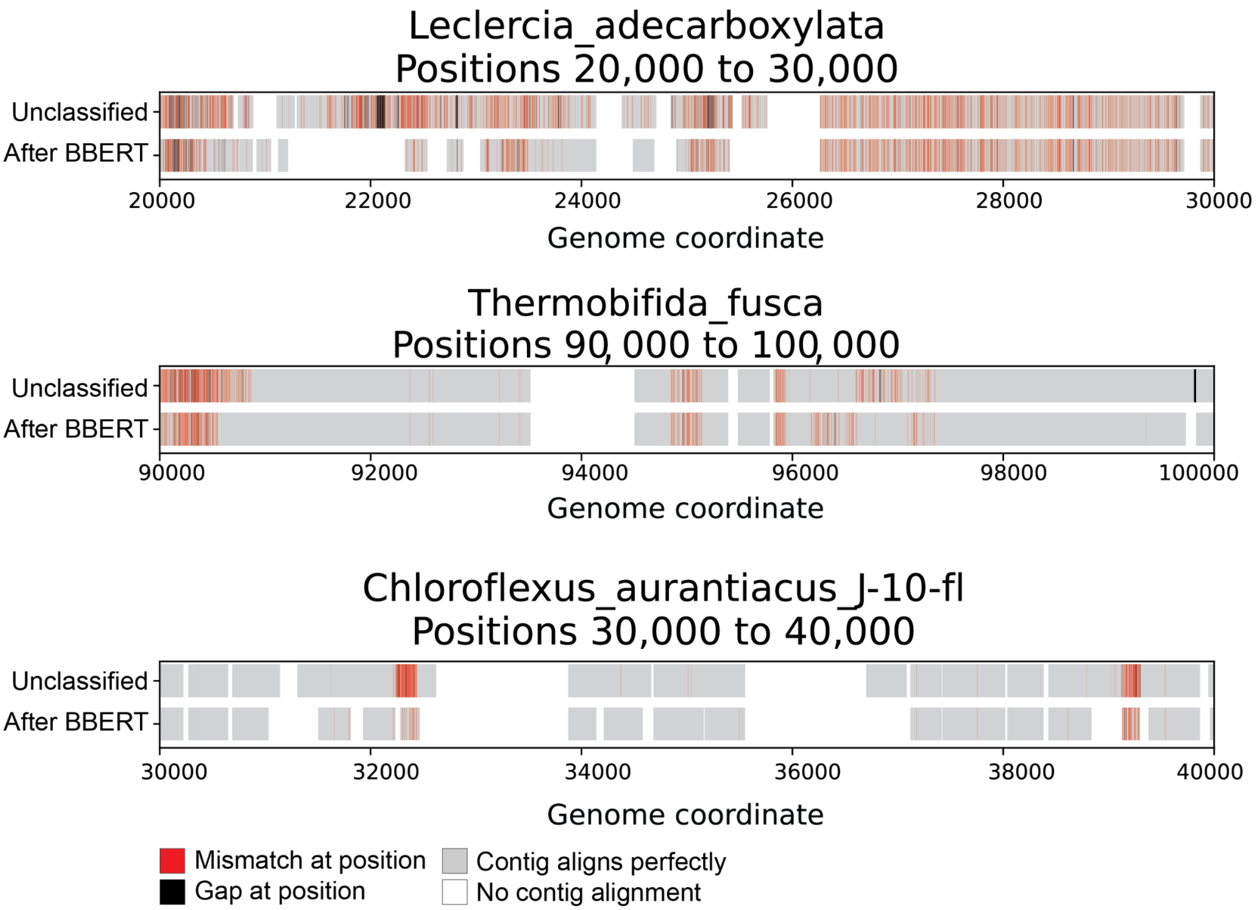

Fig. S4. Comparison of de-novo assembly errors before and after BBERT filtering.
Reads were assembled with metaSPAdes[25] using either unfiltered input or BBERT-filtered (bacterial) reads. Resulting contigs were aligned to the known bacterial source genomes used to generate the mixtures (see Methods), enabling per-base annotation of mismatches and gaps and identification of mis-assemblies. Shown are representative loci illustrating some outcomes: unfiltered data produces chimeric joins or dense local errors, whereas BBERT-filtered data assembles the region correctly or refrains from assembling (avoiding false contigs); in a minority of cases, the unfiltered assembly is correct while filtering prevents assembly, consistent with reduced coverage after removal of non-bacterial reads; some regions remain challenging under both conditions. Aggregate improvements in assembly quality and runtime are quantified in Fig. 6.

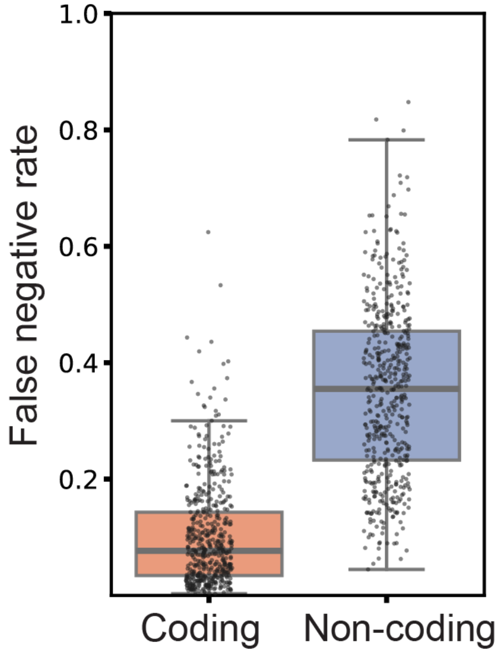

Figure S5. Comparing BBERT results on coding vs. noncoding bacterial reads. We used the four datasets of 128 bacteria genera each described in Methods Section 2.b (~140K reads).

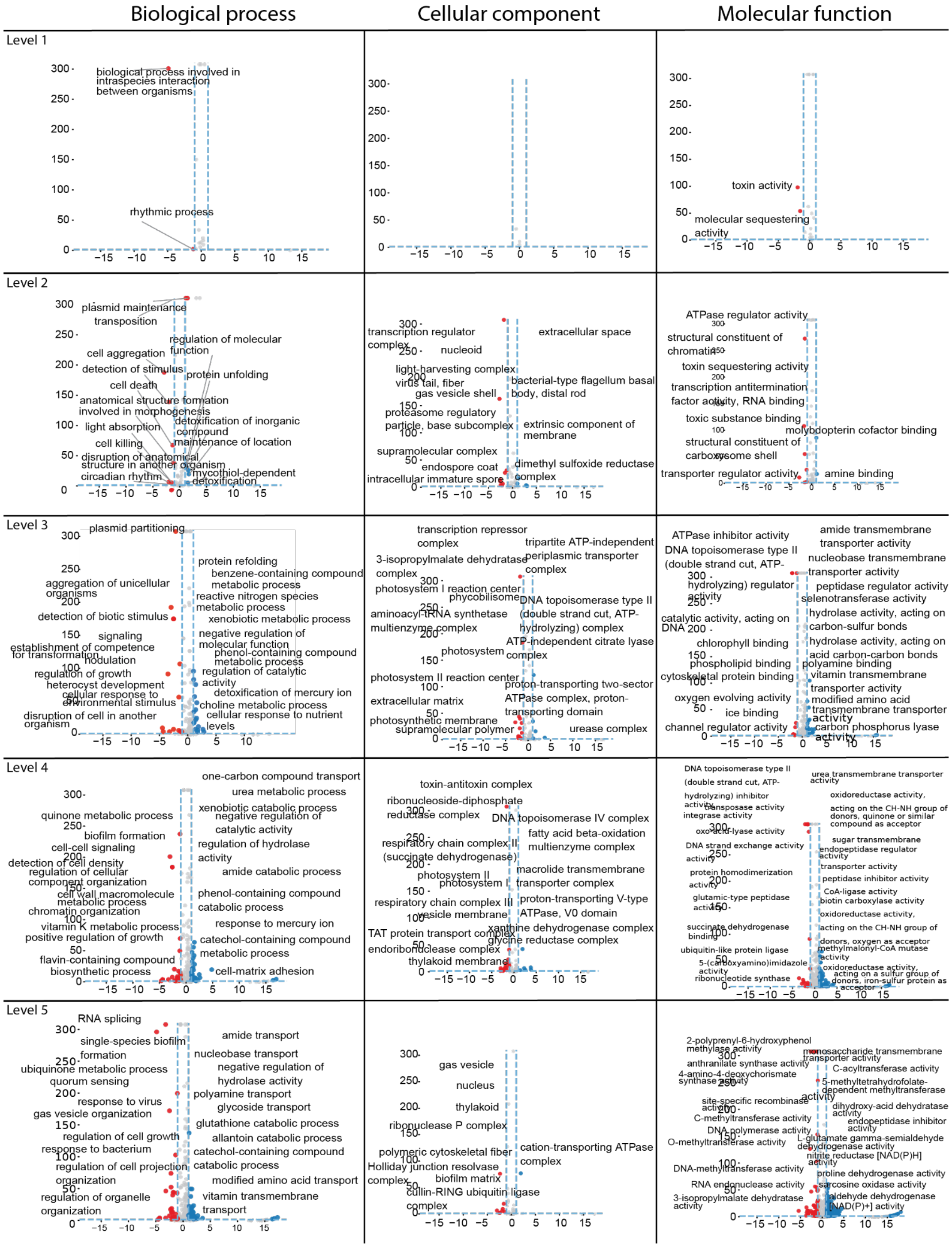

Figure S6. Gene Ontology analysis of terms enriched for False Negative and True Positive. Volcano plots for bacterial classification enrichment in true positive (blue) and false negative (red). The top 10 most significant GO terms are labeled at five GO levels (Table S9). The GO annotation process is described in the Methods section.

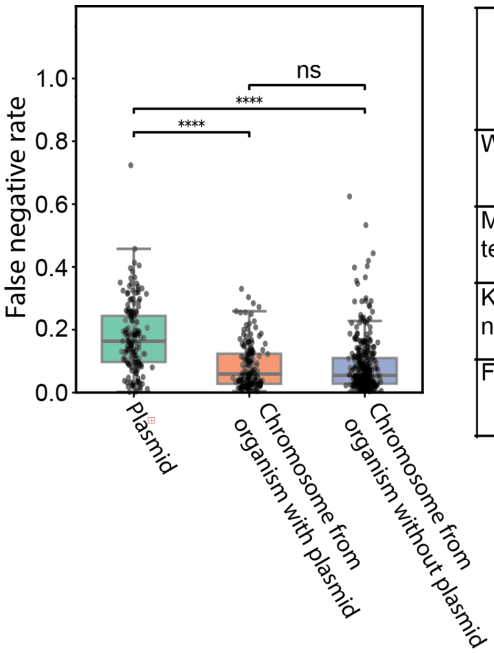

	Plasmid vs chromosome with plasmid	Plasmid vs chromosome without plasmid	Chromosome without vs chromosme with plasmid
Welch-test	p = 3.12e-17	p = 2.34e-18	p = 0.98
Mann-Whitney test	p = 1.35e-16	p = 8.00e-24	p = 0.52
Kolmogorov-Smirnov test	p = 2.16e-13	p = 4.43e-24	p = 0.41
Fisher's test	p < 1e-300<br>Odds ratio = 2.24	p < 1e-300<br>Odds ratio = 2.28	p = 2.66e-9<br>Odds ratio = 1.02

Figure S7. Comparing BBERT results on plasmid vs. non-plasmid bacterial reads. For each read, we determined whether it originated from a chromosome or a plasmid. Organisms were divided into two groups: those with at least one plasmid and those without any plasmids. Plasmids were identified based on the NCBI genome headers. We labeled the groups as follows: Plasmid, Chromosome in organisms with plasmids (ChromP), and Chromosome in organisms without plasmids (ChromN). We calculated the FN rates for each organism; statistical tests were applied to assess differences between the groups. Based on the Shapiro-Wilk normality test (Plasmid=1.574e-13, ChromP=1.755e-04, ChromN=1.522e-03), we performed group-to-group comparisons using the t-test, Mann-Whitney, and Kolmogorov-Smirnov tests. The results show a strong difference between the plasmid and non-plasmid groups, with no significant difference between chromosomal groups (Fisher test shows an Odds Ratio of 1.06). This indicates that BBERT tends to misclassify plasmid reads much more frequently than chromosomal reads, regardless of whether these chromosomal reads come from an organism that has plasmids. The significance shown is Mann-Whitney test results.

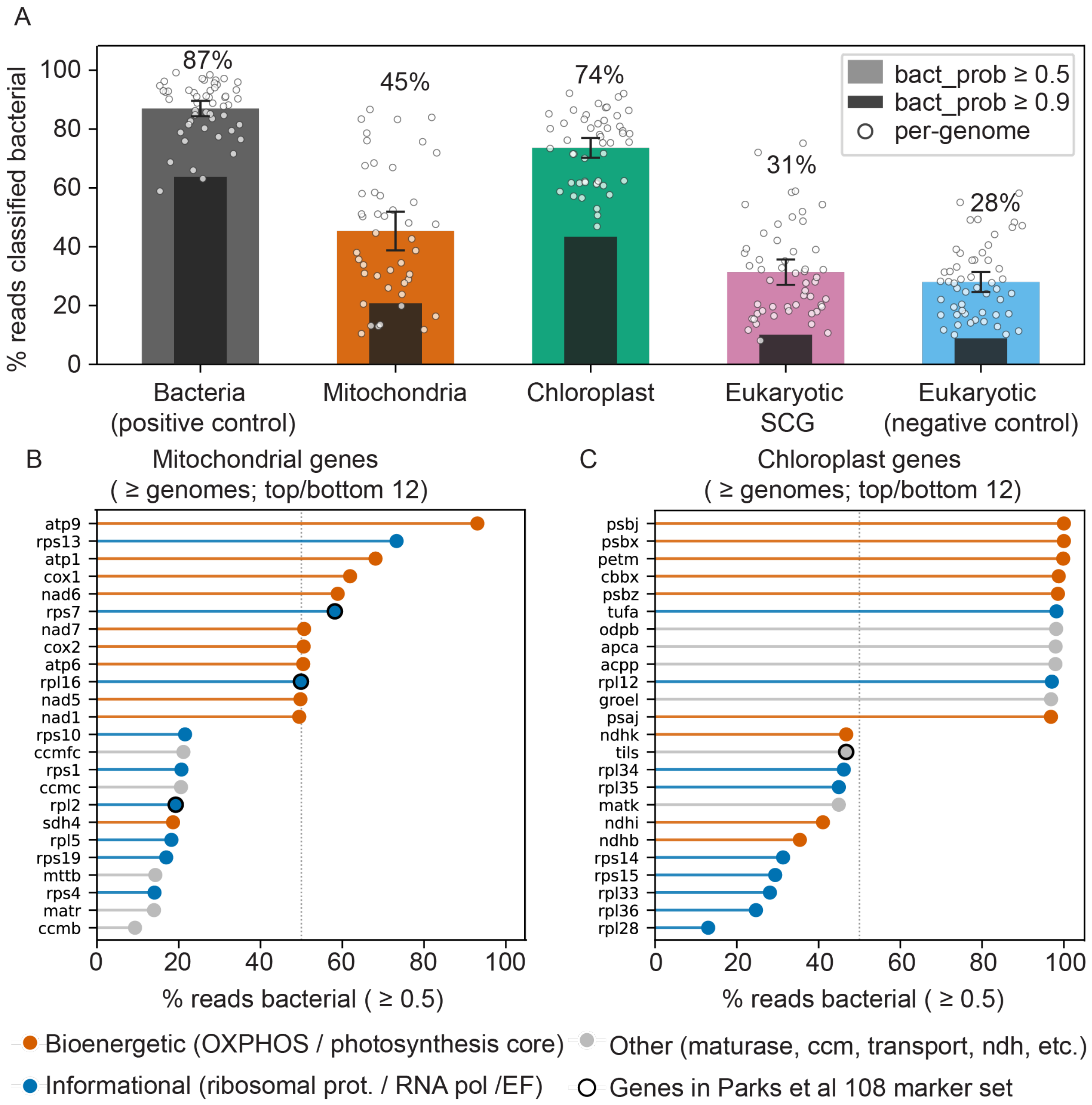

Fig. S8. Mitochondrial, chloroplast and eukaryotic single-copy genes classified as bacterial by BBERT. (A) Percentage of reads classified bacterial per category (mean across 50 genomes ± 95 % CI; grey bars, bact_prob ≥ 0.5; black overlay, ≥ 0.9; open circles, per-genome values). **(B-C)** Per-gene bacterial-classification rate (bact_prob ≥ 0.5) for **(B)** mitochondrial and **(C)** chloroplast genes present in at least 3 genomes (the 12 most- and 12 least-bacterial shown). Genes are colored by functional class: orange: bioenergetic (photosynthesis core); blue: informational (ribosomal proteins, RNA polymerase, translation factors); grey: other (maturases, cytochrome-c maturation, transport, NDH-like, ORFs). Ringed markers belong to the 108-gene Parks single-copy marker gene set used by SingleM. The conserved, endosymbiont-derived bioenergetic core classifies as bacterial most often, whereas the divergent organellar ribosomal proteins and organelle-specific accessory machinery classify as bacterial least often.
